# Direct Observation of Off‐Stoichiometry‐Induced Phase Transformation of 2D CdSe Quantum Nanosheets

**DOI:** 10.1002/advs.202205690

**Published:** 2023-01-13

**Authors:** Hyeonjong Ma, Dongjun Kim, Soo Ik Park, Back Kyu Choi, Gisang Park, Hayeon Baek, Hyocheol Lee, Hyeongseoung Kim, Jong‐Sung Yu, Won Chul Lee, Jungwon Park, Jiwoong Yang

**Affiliations:** ^1^ Department of Energy Science and Engineering Daegu Gyeongbuk Institute of Science and Technology (DGIST) Daegu 42988 Republic of Korea; ^2^ Center for Nanoparticle Research Institute for Basic Science (IBS) Seoul 08826 Republic of Korea; ^3^ School of Chemical and Biological Engineering and Institute of Chemical Processes Seoul National University Seoul 08826 Republic of Korea; ^4^ Energy Science and Engineering Research Center Daegu Gyeongbuk Institute of Science and Technology (DGIST) Daegu 42988 Republic of Korea; ^5^ Department of Mechanical Engineering BK21 FOUR ERICA‐ACE Center Hanyang University Ansan Gyeonggi 15588 Republic of Korea; ^6^ Institute of Engineering Research College of Engineering Seoul National University Seoul 08826 Republic of Korea; ^7^ Advanced Institute of Convergence Technology Seoul National University Suwon‐si Gyeonggi‐do 16229 Republic of Korea

**Keywords:** in situ transmission electron microscopy, phase transformation, quantum nanosheets, stoichiometry, two‐dimensional nanocrystals

## Abstract

Crystal structures determine material properties, suggesting that crystal phase transformations have the potential for application in a variety of systems and devices. Phase transitions are more likely to occur in smaller crystals; however, in quantum‐sized semiconductor nanocrystals, the microscopic mechanisms by which phase transitions occur are not well understood. Herein, the phase transformation of 2D CdSe quantum nanosheets caused by off‐stoichiometry is revealed, and the progress of the transformation is directly observed by in situ transmission electron microscopy. The initial hexagonal wurtzite‐CdSe nanosheets with atomically uniform thickness are transformed into cubic zinc blende‐CdSe nanosheets. A combined experimental and theoretical study reveals that electron‐beam irradiation can change the stoichiometry of the nanosheets, thereby triggering phase transformation. The loss of Se atoms induces the reconstruction of surface atoms, driving the transformation from wurtzite‐CdSe(11$\bar{2}$0) to zinc blende‐CdSe(001) 2D nanocrystals. Furthermore, during the phase transformation, unconventional dynamic phenomena occur, including domain separation. This study contributes to the fundamental understanding of the phase transformations in 2D quantum‐sized semiconductor nanocrystals.

## Graphical abstract

This study reveals the mechanism for phase transformation of 2D CdSe quantum nanosheets induced by off‐stoichiometry. The loss of Se atoms in 2D wurtzite‐CdSe nanocrystals destabilizes the initial structure and modifies the surface atomic configuration to resemble zinc blende structure, which ultimately leads to phase transformation. Additional non‐conventional dynamic phenomena, including domain separation, are observed during the transformation.

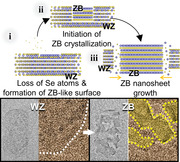

## 1 Introduction

The crystal structures of solid‐state materials, which are defined by periodic arrangements of repeating units, determine their electronic band structure,^[^ ^1^, ^2^ ^]^ mechanical characteristics,^[^ ^3^ ^]^ and other physical properties.^[^ ^4^, ^5^, ^6^ ^]^ From this perspective, crystal phase transitions offer an important pathway to control the intrinsic characteristics of solid materials in working devices such as memory devices^[^ ^7^, ^8^, ^9^, ^10^, ^11^ ^]^ and sensors.^[^ ^12^, ^13^ ^]^ The energy barrier to phase transitions decreases as the dimension of a crystal material decreases;^[^ ^14^, ^15^, ^16^, ^17^ ^]^ consequently, phase transformations are more likely to occur in small nanocrystals. However, a fundamental mechanistic understanding of nanocrystal phase transformations is lacking. Even though there have been studies based on conventional characterization methods such as in situ X‐ray diffraction (XRD) and optical spectroscopy,^[^ ^18^, ^19^, ^20^ ^]^ important aspects of the nanocrystal phase transformations are easily lost in such studies. This is mainly because the phase transformations at the nanoscale progress in an asynchronous manner, whereas the typical measurements methods mentioned above are based on ensemble‐averaged information. Therefore, direct microscopic observation, which can resolve individual participating pathways during dynamic phase transformations, would be an ideal approach to understand the processes involved. In situ transmission electron microscopy (TEM) provides an opportunity to examine the individual pathways of structural changes at the atomic scale either in liquid^[^ ^21^, ^22^, ^23^, ^24^ ^]^ or solid‐state.^[^ ^25^, ^26^, ^27^, ^28^, ^29^ ^]^ There are a few studies that have employed this method to explore the phase transformations.^[^ ^30^, ^31^, ^32^, ^33^, ^34^, ^35^, ^36^, ^37^ ^]^ Nonetheless, in most cases, only exemplary events that are likely controlled by temperature^[^ ^30^, ^31^, ^32^, ^33^ ^]^ and electricity^[^ ^34^, ^35^, ^36^, ^37^ ^]^ are reported. It has been challenging to clearly identify physical or chemical factors that control the phase transitions and describe their roles in observed pathways.

Quantum‐sized semiconductor nanocrystals have attracted considerable attention owing to their unique properties, including size‐ and shape‐dependent electronic band structure, bright emission with high color purity, and effective light absorption.^[^ ^38^, ^39^, ^40^ ^]^ In addition, recent studies have revealed that minor changes in the atomic arrangement of quantum dots can significantly alter their properties.^[^ ^41^, ^42^, ^43^ ^]^ CdSe, a II–VI compound semiconductor, is the most representative semiconductor nanocrystal because of their bandgap tunability (i.e., over the entire visible range) and well‐developed methods for size‐^[^ ^44^, ^45^ ^]^ and shape‐controlled^[^ ^46^, ^47^, ^48^, ^49^, ^50^ ^]^ synthesis. In addition, it is well‐known that CdSe has several crystal structures, the most common being hexagonal wurtzite and cubic zinc blende phases.^[^ ^44^, ^45^, ^46^, ^47^, ^48^, ^49^, ^50^ ^]^ Several studies have suggested that the crystal structure of CdSe nanocrystals can be tuned depending on the experimental conditions, such as pressure, strain, temperature, and chemical composition of synthetic solvent.^[^ ^51^, ^52^, ^53^, ^54^, ^55^, ^56^ ^]^ However, important events that occur in CdSe nanocrystals and their effect on the progress of the phase transformation are poorly understood, necessitating high‐resolution in situ observations at a level that clearly capture changes in the CdSe crystal domains.

Herein, using high‐resolution in situ TEM, we reveal the phase transformation of 2D CdSe quantum nanosheets induced by their off‐stoichiometry. CdSe quantum nanosheets with a wurtzite structure are transformed into a zinc blende structure via dynamic atomic movements. The phase transformation is initiated upon the loss of Se atoms, destabilizing 2D wurtzite‐CdSe nanosheets. This results in the modification of the surface atomic configuration, causing it to resemble the structure of zinc blende. Notably, the phase transformation can also be regulated by controlling the reaction temperature, implying that the temperature and stoichiometry of nanocrystals are important factors determining the crystal phase. During the phase transformation, domain separation and coalescence processes occurred, suggesting that the nanocrystal phase transformations occur via dynamic and collective atomic motions, even in the solid‐state.

## 2 Results and Discussion

In this study, ultrathin 2D CdSe quantum nanosheets (thickness ≈ 1.4 nm)^[^ ^57^ ^]^ were selected as a model material and prepared by colloidal synthesis to investigate the phase transformation behavior. 2D nanosheets are ideal materials for investigating phase transformations, because the dynamic changes can be easily observed by TEM owing to their 2D shape and ultrathin thickness. Since TEM only shows 2D projected images of the sample, understanding the transformation of thicker materials is not straightforward; if there are many atomic layers in the direction of electron‐beam transmission, the dynamic changes that occur within the nanocrystal will overlap in the TEM observations. In contrast, most of the atoms in 2D nanocrystals are exposed in TEM observation because of the limited number of atomic layers in the thickness direction. Furthermore, the large lateral size of 2D nanocrystals is beneficial for observing dynamic processes by TEM, because in such nanocrystals, a large projected area is available for observation.

**Figure 1:**
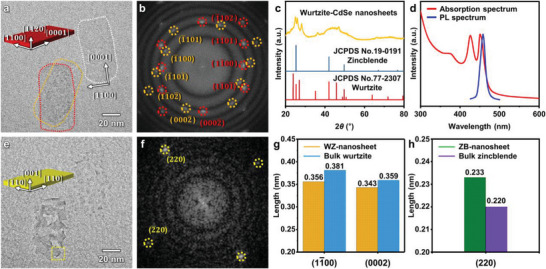
Phase transformation of the wurtzite‐CdSe nanosheets. a) TEM image of the 2D CdSe quantum nanosheets before phase transformation and b) corresponding FFT pattern. c) XRD pattern and d) absorption and PL spectra of the wurtzite‐CdSe nanosheets. e) TEM image of the 2D CdSe quantum nanosheets after phase transformation and f) the FFT pattern of the boxed area. g,h) Measured d‐spacings of the CdSe quantum nanosheets with g) wurtzite and h) zinc blende crystal structures in comparison with those of bulk crystals.

TEM images of the as‐synthesized nanosheets are shown in **Figure** **1**a. The nanosheets comprise 2D structures with lateral size of ≈80 nm × 40 nm and uniform thicknesses of 1.4 nm (Figures S1 and S2, Supporting Information). The lateral dimension of the nanocrystals is much larger than the exciton Bohr radius of CdSe (5.4 nm),^[^ ^58^ ^]^ while the thickness is much smaller. The observed morphology defines the synthesized nanocrystals as quantum‐sized 2D nanocrystals. The TEM image and the corresponding fast Fourier transform (FFT) pattern (Figure 1b) indicate that the nanocrystals have a wurtzite crystal structure, which is also confirmed by XRD data (Figure 1c). Absorption and photoluminescence (PL) spectra show that the optical bandgap of the nanosheets is 2.7 eV (Figure 1d), implying strong quantum confinement because of the ultrathin thickness (the bandgap of bulk CdSe is 1.7 eV). The observed bandgap matches well with the reported value for 2D wurtzite‐CdSe nanocrystals with thicknesses of 1.4 nm (corresponding to ≈7 atomic monolayers).^[^ ^57^ ^]^ The peaks in both spectra are extremely sharp (full width at half maximum of ≈15 nm for PL emission), which is attributed to the uniform thickness of the 2D nanocrystals. In addition, the absorption spectrum exhibits the splitting of heavy‐hole/electron and light‐hole/electron transitions, representative characteristic of 2D Cd‐chalcogenide nanocrystals.^[^ ^48^, ^49^, ^57^, ^59^, ^60^ ^]^

Upon exposure to the electron beam, the crystal structure of the nanosheets changed from wurtzite to zinc blende (See Figure S3, Supporting Information to compare their crystal structures), as shown by the in situ TEM data and representative TEM images (Figure 1a,e and Movie S1, Supporting Information). The lack of wurtzite spots in the FFT pattern after the transformation confirms a complete transition from wurtzite to zinc blende (Figure 1f; see Figure S4, Supporting Information, for the FFT pattern acquired from the entire image). It is well known that the d‐spacings (especially in the lateral direction) of 2D CdSe nanocrystals are different from those of bulk CdSe crystals and 0D CdSe nanocrystals by ≈5%, because of the high surface tension along their atomically flat basal planes (Figure 1g,h and Tables S1 and S2, Supporting Information).^[^ ^49^, ^57^, ^60^ ^]^ The observed d‐spacing of the (220) plane of zinc blende CdSe crystals in the lateral direction is 0.233 nm, which is much larger than that of bulk zinc blende CdSe crystals (0.220 nm) and 0D CdSe nanocrystals (0.215 nm).^[^ ^61^ ^]^ Instead, the observed d‐spacing is consistent with the reported values for the lateral (220) plane of 2D zinc blende CdSe nanocrystals with thicknesses of ≈2 nm (Table S2, Supporting Information).^[^ ^60^ ^]^ Thus, this strongly supports that the newly formed nanocrystals have a 2D zinc blende structure. Moreover, all of these nanocrystals are aligned to show their CdSe(001) basal planes (Figure S4, Supporting Information for the corresponding FFT pattern), which further verifies their morphology.

Notably, the shape of the zinc blende CdSe nanosheets is different from that of the initial wurtzite nanosheets. Each wurtzite nanosheet turned into dozens of 2D zinc blende structures with a reduced lateral size. Scanning TEM (STEM) and energy‐dispersive X‐ray spectroscopy (EDS) elemental mapping images of the CdSe nanosheets before and after electron‐beam irradiation show that the wurtzite and zinc blende nanosheets have different appearances, while they retain the same constituent atoms (Figure S5, Supporting Information). The mechanisms by which the lateral size and shape change during the phase transformation are discussed later.

**Figure 2:**
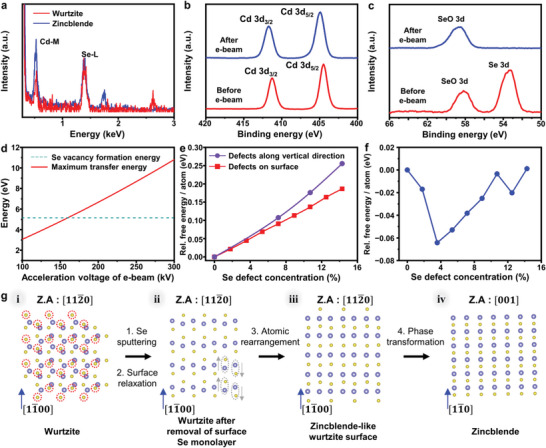
Stoichiometry change in the CdSe quantum nanosheets upon electron‐beam irradiation. a) EDS (acquired from Figure S5, Supporting Information), b) Cd XPS, and c) Se XPS profiles of the CdSe nanosheets before and after electron‐beam irradiation. d) The calculated maximum energy transferred from the electron beam to Se atoms as a function of the accelerating voltage. The dotted line represents the Se vacancy formation energy for the wurtzite‐CdSe nanosheets. e) Relative free energy per atom in the wurtzite‐CdSe nanosheets (7 atomic monolayers) as a function of the number of Se defects on the surface of basal planes (corresponding to wurtzite‐CdSe$\left(11\bar{2}0\right)$ surfaces) and along the vertical direction (corresponding to wurtzite‐CdSe [11$\bar{2}$0] direction). f) Relative free energy per atom in the Cd‐excess zinc blende CdSe nanosheets (7 atomic monolayers) as a function of the number of Se defects on the basal plane (corresponding to zinc blende CdSe(001) surfaces). For the panels (e) and (f), the Se defect concentration is defined as the number of Se defects/total number of Se atoms before the defect formation. g) Atomic configuration on the surface of 2D nanocrystals during phase transformation process. The atoms with blue and yellow colors represent Cd and Se atoms, respectively. The red dotted circles represent Se atoms to be removed.

We analyzed the stoichiometry of the CdSe nanosheets before and after the phase transformation through EDS to understand the underlying mechanism of the phase transformation from wurtzite to zinc blende (**Figure** **2**a). The EDS spectra were acquired from the initial wurtzite‐CdSe and the zinc blende‐CdSe nanocrystals in Figure S5 (Supporting Information). The atomic fraction of Cd and Se atoms in the nanosheets changes during the phase transformation. The average Cd:Se atomic ratio is 1:1 for the wurtzite‐CdSe nanosheets, which corresponds to the stoichiometric composition. However, after the phase transformation, Cd:Se atomic ratio become ≈1:0.8 for the zinc blende‐CdSe nanocrystals, implying the loss of Se atoms during the phase transformation.

Ex situ characterization was conducted using electron‐beam surface‐treatment equipment (CEBI‐200‐2250) to gain additional insight into the phase transformation upon electron‐beam irradiation. The wurtzite‐CdSe nanosheets were treated by electron‐beam irradiation at an accelerating voltage of 200 kV and an operating current of 10 mA for 10 min (Experimental Section and Figure S6, Supporting Information). The CdSe nanosheets were characterized by X‐ray photoelectron spectroscopy (XPS) before and after electron‐beam irradiation (Figure 2b,c). The Cd XPS profiles before and after irradiation both show two main peaks corresponding to Cd 3d_5/2_ and Cd 3d_3/2_. The Se XPS spectrum before irradiation exhibits two peaks corresponding to Se 3d and SeO 3d. The SeO peak is attributed to the partial surface oxidation of the CdSe nanosheets and residue of the excess Se precursors. After irradiation, the Se 3d peak diminishes, suggesting that the Se atoms located near the surface are removed. In addition, the XRD peaks have a reduced intensity and broader width after irradiation (Figure S7, Supporting Information), which is attributed to the transformation to a less crystalline structure by the loss of Se atoms. The ex situ characterization also reveals the partial detachment of surface ligands upon electron‐beam irradiation, which presumably occurred prior to the loss of Se atoms. Thermogravimetric analysis (TGA) of the nanosheets before and after electron‐beam irradiation reveals that the mass fraction of surface ligands decreases from 38 to 20 wt.% (Figure S8, Supporting Information). These results demonstrate that electron‐beam irradiation results in the removal of surface Se atoms and partial detachment of ligands, resulting in the formation of less crystalline structures.

The phase transformation process was further investigated by density functional theory (DFT) calculations (Figure 2d–g). The 2D wurtzite and zinc blende CdSe nanocrystal models used for calculations are illustrated in Figures S9–S11 (Supporting Information). The structures resemble the experimentally observed ones with regard to the thickness and crystallographic direction. The maximum energy transferable from the 200 keV electron beam to the Se atoms is ≈6.64 eV (Figure 2d, 10.81 eV for 300 keV electron beam),^[^ ^62^ ^]^ which is higher than the Se vacancy formation energy for the wurtzite‐CdSe nanosheets (5.12 eV). This indicates that the Se atoms can be sputtered from the nanocrystals by the electron‐beam irradiation. We calculated the free energy per atom of wurtzite‐CdSe nanosheets (7 atomic monolayers thick) for different numbers of Se defects on the surface of basal plane (Figure 2e). The calculated free energy per atom increases with the number of Se defects, suggesting that the wurtzite phase becomes thermodynamically unstable as Se atoms are removed from non‐polar wurtzite‐CdSe$\left(11\bar{2}0\right)$ surface (Figures S9 and S10, Supporting Information, show the relaxed structures of the wurtzite‐CdSe nanosheets with Se defects along the crystal surface and vertical direction, respectively). Considering the interaction of the electron beam with the atoms of the crystal and the results of DFT calculations (Figure 2e), the loss of deeper‐layer Se atoms is not highly probable. Contrarily, the free energy per atom of the zinc blende 2D crystals with Se defects (7 atomic monolayers in the thickness) is even lower than that of zinc blende 2D crystals without Se defects (Figure 2f), which confirms zinc blende structure can be maintained while the certain level of Se defects are introduced into the crystal. The loss of surface Se atoms destabilizes wurtzite nanosheets with non‐polar basal planes (corresponding to wurtzite‐CdSe$\left(11\bar{2}0\right)$ surfaces), while it can stabilize zinc blende nanosheets with polar basal planes (corresponding to zinc blende‐CdSe(001) surfaces). This is consistent with the results of a previous study showing that the basal planes of zinc blende 2D CdSe nanocrystals are typically found in Cd‐rich states.^[^ ^60^ ^]^

DFT calculations also provide clues on changes in the atomic arrangement that might occur during the phase transformation. The atomic arrangements of the surface atoms on the basal planes during the phase transition from the initial 2D wurtzite nanocrystals to 2D wurtzite nanocrystals with Se defects, and to 2D zinc blende nanocrystals, are displayed in Figure 2g. The side views and 3D configurations are provided in Figure S12 (Supporting Information). After removal of surface Se monolayers, the arrangement of surface atoms obtained from the relaxed structure (Figure S9i, Supporting Information) is between that of the wurtzite‐CdSe$\left(11\bar{2}0\right)$ and the zinc blende‐CdSe(001) 2D nanocrystals surfaces. Thus, the calculation results indicate that the change in stoichiometry can cause changes in surface atomic configurations, which can then lead to the phase transformation. The atomic ratio of Cd:Se calculated from the suggested theoretical model (Cd:Se = 1:0.85 and 1:0.7 after the loss of surface Se monolayer from the single and both sides of basal planes, respectively) matches that acquired from EDS analysis on the zinc blende nanocrystals (Figure 2a). Furthermore, the suggested mechanism is consistent with previous works showing the formation of the less crystalline intermediates during the solid‐to‐solid transformation of other solid materials.^[^ ^63^, ^64^ ^]^

Usually, the phase transformation is a process leading to the formation of a stable structure. This process is likely to be controlled by other reaction conditions such as the crystal size^[^ ^65^ ^]^ and the temperature.^[^ ^31^, ^32^, ^66^, ^67^ ^]^ First, the effect of the thickness of nanosheets on the phase transformation was investigated (Figure S13, Supporting Information). Thicker wurtzite‐CdSe nanosheets (8 atomic monolayers in the thickness) showed the partial phase transformation with a slower rate of transformation, compared to CdSe nanosheets with the thickness of 7 atomic monolayers. We investigated the effects of temperature on the phase transformation by heating the sample during in situ TEM (Figure S14, Supporting Information). The results show that the phase transformation from wurtzite to zinc blende occurred below 200 °C; however, it did not occur at 300 °C, even after electron‐beam irradiation for ≈600 s. The TEM images at 300 °C are similar to those prior to electron‐beam irradiation regardless of the duration of the electron‐beam irradiation. In general, atomic movements become more active at high temperature, as atoms in the lattice can have enough thermal energy. However, our experiment shows that the phase transformation is suppressed at higher temperatures, supporting that the transition is thermodynamically controlled. This result is consistent with that of previous studies showing that the wurtzite phase is more stable than the zinc blende phase at the high temperature condition.^[^ ^68^ ^]^

Ex situ heating experiments were conducted to better understand the effect of temperature by excluding the effect of stoichiometry changes. The wurtzite‐CdSe nanosheets were deposited on a glass substrate and annealed for 30 min at a wide range of temperatures. The XRD data of the nanosheets annealed at 50–200 °C are almost identical to that of the initial sample (Figure S15, Supporting Information). When annealed at 300 °C, the XRD pattern of the sample shows sharp peaks and their positions correspond to bulk wurtzite crystals, implying that some of the nanosheets aggregate to form large crystals at 300 °C. This result is consistent with the TEM images of the wurtzite‐CdSe nanosheets after annealing (Figure S16, Supporting Information). When the annealing temperature is >200 °C, TEM images show that some of the nanosheets aggregate to form nanocrystals having bulk‐like d‐spacings (the d‐spacing of the (0002) plane of the CdSe crystal is 0.359 nm in Figure S16d, Supporting Information). These results clearly demonstrate that heating, by itself, does not induce the phase transformation of the nanosheets.

**Figure 3:**
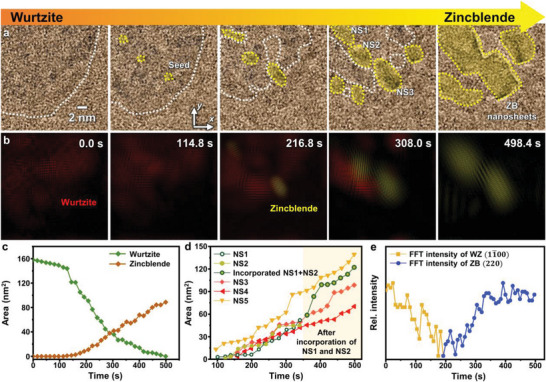
In situ high‐resolution TEM analysis of the phase transformation. a) Time‐series TEM images and b) inverse‐FFT images showing the phase transformation. White and yellow dashed lines represent wurtzite and zinc blende boundaries, respectively. See Movie S2 (Supporting Information for the corresponding video). Time‐dependent area changes of c) wurtzite and zinc blende regions and d) five selected zinc blende nanosheets (NS1–NS5), as highlighted in the fourth panel of Figure 3a and Figure S18 (Supporting Information). e) Time‐dependent changes of the FFT intensity of wurtzite $\left(1\bar{1}00\right)$ and zinc blende (220), acquired from the region corresponding to NS3 in the panel (a).

The dynamic changes of the nanocrystals during the phase transformation were observed by high‐resolution in situ TEM (Movie S2, Supporting Information). In the atomic‐resolution snapshots of the process (**Figure** **3**a), the wurtzite‐CdSe nanosheets are highlighted by white dashed lines and the zinc blende phases are highlighted in yellow. The corresponding inverse‐FFT images show the dynamic changes in the crystal structure more clearly (Figure 3b). The area of the wurtzite phase decreases as that of the zinc blende phase increases (Figure 3c), supporting the phase transformation. Notably, one initial wurtzite nanosheet is transformed into multiple zinc blende crystals with considerably different lateral shapes, which is attributed to the two following effects. First, the phase transformation initiates from the multiple sites of the initial wurtzite‐CdSe nanosheets, because the loss of Se atoms occurs simultaneously at the multiple locations. Second, the overall lateral size of crystals is decreasing during the phase transformation, which is also confirmed by Figure 3c. Owing to the difference in crystal structure between the wurtzite and zinc blende phases, 2D crystals with the same number of atomic monolayers have different morphologies. For example, wurtzite crystals with seven atomic monolayers have a thickness of ≈1.4 nm, whereas zinc blende crystals with the same number of atomic monolayers are ≈2 nm thick. In other words, zinc blende crystals might have reduced lateral size compared to the wurtzite crystals with the same number of atoms. Furthermore, some Cd atoms may be removed from the nanosheets during electron‐beam irradiation, which is supported by the DFT calculations showing that the loss of Cd atoms from the wurtzite nanosheets becomes thermodynamically favorable for the nanosheets with the high Se defect concentration (Figure S17, Supporting Information). Consequently, during the phase transformation, multiple zinc blende domains are formed simultaneously with the shrinkage of the overall crystalline area, inducing the crystal domain separation discussed later. This results in the formation of multiple zinc blende nanosheets from a single wurtzite nanosheet.

The growth trajectories of five individual zinc blende nanosheets (denoted as NS1–NS5) are examined in detail (Figure 3d). NS1–NS3 are highlighted in Figure 3a. NS4 and NS5 are selected from the additional in situ TEM data shown in Figure S18 (Supporting Information; see Movies S3 and S4, Supporting Information, for the corresponding video). Most of the zinc blende seeds appear in a short time range (≈100–150 s) and their growth tendency is similar except the coalescence between NS1 and NS2. This coalescence process is discussed in more detail later. Interestingly, the zinc blende phases are seeded from sites at which the wurtzite lattice fringes disappear. This is clearly verified by the time‐dependent changes of the FFT intensity of wurtzite $\left(1\bar{1}00\right)$ and zinc blende (220), acquired from the region corresponding to NS3 (Figure 3e). The phase transformation initiates in areas where the wurtzite crystals become less crystalline owing to the loss of Se atoms, which is consistent with DFT calculations. Changes to the crystal orientation of four individual zinc blende nanosheets (NS2–NS5) are analyzed in Figure S19 (Supporting Information). The orientation of NS1 could not be analyzed because the lattice fringe was not revealed until it coalesced with NS2. During the growth of the zinc blende domains, the crystal orientation remains almost constant. This suggests that the locations of initially formed zinc blende crystals are fixed, and that the zinc blende phases propagate from the initial seed parts.

**Figure 4:**
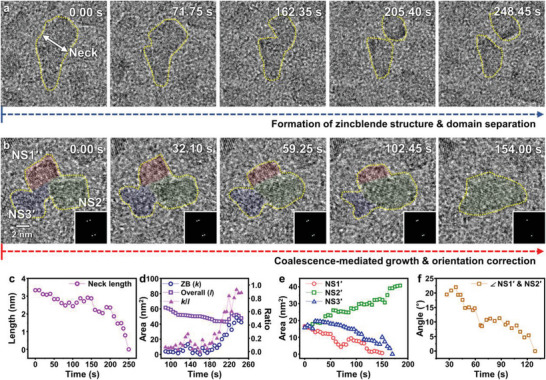
Unconventional dynamic phenomena during the phase transformation. Time‐series TEM images showing a) domain separation and b) coalescence with orientation correction during the phase transformation. See Movies S5 and S6 (Supporting Information) for corresponding videos of (a) and (b), respectively. c) Time‐dependent change in the neck length. The location of the neck is shown in the first panel of (a). d) Time‐dependent changes in the zinc blende area (*k*), overall particle area (*l*, defined as the projected area highlighted by yellow dashed line in the panel (a)), and their ratio (*k*/*l*). e) Time‐dependent area changes of NS1′, NS2′, and NS3′ (highlighted by red, green, and blue in the panel (b), respectively). f) Angle between [220] directions of NS1′ and NS2′ as a function of time.

In situ TEM also revealed several non‐classical phenomena involved in the phase transformation (**Figure** **4** and Movies S5 and S6, Supporting Information), demonstrating that active atomic movements affect the phase transformation of nanocrystals occurring in the solid‐state. In particular, domain separation (Figure 4a) and coalescence (Figure 4b) of the zinc blende nanosheets were observed. Strikingly, the separation of the crystal domains occurs during the phase transformation, as shown in Figure 4a and Movie S5 (Supporting Information; See the schematic illustration of this process in Figure S20, Supporting Information). An initial nanosheet in Figure 4a shows less crystalline features because of the loss of Se atoms. The phase transformation initiates from the initial domain, forming two zinc blende nanosheets. During this process, the neck diameter decreases over time, while the two crystalline domains steadily separate from each other (Figure 4c). This implies that the atoms located between the two crystalline domains are diffused to either side of the neck to form two stable zinc blende structures. After the separation of the nanosheet with less crystallinity into two domains, the new domains show strong lattice fringes corresponding to zinc blende crystals (Figure 4d). This demonstrates that crystal domain separation is accompanied by the re‐arrangement of atoms to form zinc blende crystals. This is consistent with the underlying mechanism of the formation of multiple zinc blende crystals from a single wurtzite nanosheet, which is discussed in the above part on Figure 3. To the best of our knowledge, the crystal domain separation process has not been observed previously, representing the uniqueness of the observed phase transformation.

In addition, the coalescence of zinc blende nanosheets was observed (Figure 4b and Movie S6, Supporting Information). In Figure 4b, three zinc blende crystal domains are represented as NS1′–NS3′, which are highlighted in red, green, and blue, respectively. The time‐dependent area changes of these domains are plotted in Figure 4e. The areas of NS1′ and NS3′ decrease as that of NS2′ increases, suggesting that NS1′ and NS3′ are incorporated into NS2′. Finally, these nanosheets merge into a single larger nanosheet, as shown in the last panel of Figure 4b. The difference in crystal orientation between the NS1′ and NS2′ domains (based on [220]) was measured during the coalescence process (Figure 4f). Initially, the relative misorientation between the [220] directions of NS1′ and NS2′ is ≈19.5°. The orientation difference between the two domains decreases over time until reaching 0° at 130 s. A time‐series TEM images in the time range of 0–166.95 s provides more detailed views of the crystal orientation realignment during the coalescence process (Figure S21, Supporting Information). Orientation correction during coalescence is attributed to the relaxation of strain upon matching the crystallographic direction between NS1′ and NS2′ to form a thermodynamically stable structure.^[^ ^69^ ^]^ Notably, such large orientation changes were not observed for zinc blende nanocrystals that did not coalesce. The oriented attachment during the coalescence of nanocrystals has been usually observed during solution‐phase reactions.^[^ ^70^, ^71^, ^72^, ^73^, ^74^ ^]^ Our observations clearly show that similar phenomena can occur in the solid‐state.^[^ ^75^ ^]^ These dynamic atomic movements that allow the formation of stable structures provide strong evidence that not only atomic displacement within a crystal but also atomic movement between crystals is important mechanisms underlying this phase transformation.

## 3 Conclusion

**Figure 5:**
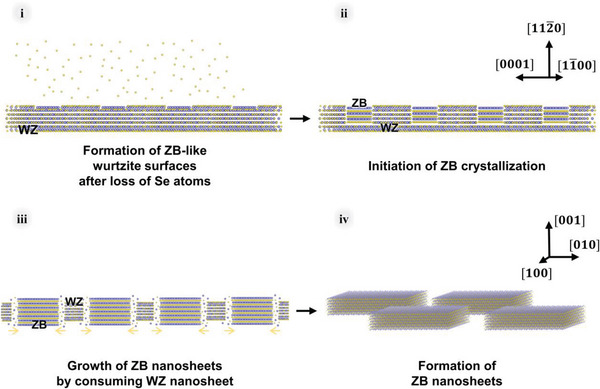
Schematic of the suggested phase transformation mechanism. The atoms with blue and yellow colors represent Cd and Se atoms, respectively.

We investigated the phase transformation of 2D CdSe semiconductor quantum nanosheets. From our experimental and theoretical results, the mechanism of the phase transformation is as follows (**Figure** **5**). i) The loss of Se atoms results in off‐stoichiometry. Parts of the nanosheets become less crystalline and exhibit novel surface atomic configurations that are intermediate between that of the wurtzite‐CdSe$\left(11\bar{2}0\right)$ and zinc blende‐CdSe(001) 2D nanocrystal basal planes. ii) The formation of zinc blende crystals are initiated from the multiple sites of less crystalline parts (presumably due to the loss of Se atoms) on the wurtzite nanosheets. iii) The zinc blende crystals are propagated by consuming the original wurtzite crystals, resulting in the domain separation. iv) Multiple zinc blende‐CdSe nanosheets are formed.

Atomic‐scale imaging allowed us to investigate the reaction process accompanied by dynamic phenomena by observing the changes in the lattice fringes. Diverse pathways were revealed during the phase transformation, such as domain separation and coalescence. Direct observation of the phase transformation showed that dynamic atomic movements occur. Thus, the phase transformation was accompanied with atomic displacement within the crystal as well as atomic movement between crystals.

Our findings provide a fundamental understanding of phase transformations in quantum‐sized 2D semiconductors and introduce a new opportunity for controlling the crystal phase. We envision that the observed phase transformation of 2D semiconductor nanocrystals will facilitate the application of such nanocrystals in systems wherein precise spatial control of the phase transition is required. For example, high‐resolution patterning of the crystal phase will be possible by targeted electron‐beam irradiation. This cannot be achieved by conventional heat‐induced phase transformations, because heating cannot be applied with high spatial resolution.

## 4 Experimental Section

### Materials

Cadmium(II) chloride (CdCl_2_, 99.99%), octylamine (OcAm, 99%), oleylamine (OAm, 70%), trioctylphosphine (TOP, 97%), selenium (Se, 99.99%), ammonium persulfate (≥98%), and chloroform (99%) were purchased from Sigma–Aldrich. 1‐Butanol (99%) was purchased from Samchun Chemical Co. TEM grids were purchased from Electron Microscopy Sciences.

### Synthesis of Wurtzite‐CdSe Quantum Nanosheets

Wurtzite‐CdSe quantum nanosheets were prepared following a previously reported method^[^ ^57^ ^]^ with minor modifications. In brief, CdSe nanosheets (7 atomic monolayers in the thickness) were synthesized by reacting a CdCl_2_(OcAm,OAm)_2_ complex with elemental Se. The CdCl_2_(OcAm,OAm)_2_ complex solution was prepared by heating a mixture of CdCl_2_ (1.5 mmol), OcAm (5.0 ml), and OAm (5.0 ml) at 120 °C for 2 h. The Se precursor solution was prepared by stirring elemental Se (4.5 mmol) in a mixture of OcAm (2.5 ml) and OAm (2.5 ml). The Se precursor solution (5.0 ml) was introduced into the CdCl_2_‐ammine complex solution at room temperature (25 °C). Then, the mixture was heated to 100 °C and held for 16 h. Thicker nanosheets (8 atomic monolayers in the thickness) were obtained when the reaction time was extended to 32 h (Figure S13, Supporting Information). The nanosheets were precipitated with 1‐butanol and TOP and purified by a standard centrifugation method using 1‐butanol and chloroform. The produced nanosheets were re‐dispersed in chloroform for further study.

### In Situ TEM Analysis

The CdSe nanosheets were loaded on TEM grids with amorphous carbon film by dropping a few drops of a chloroform solution containing the nanosheets. To analyze the phase transformation, in situ TEM and STEM/EDS of the nanosheets were performed with a Titan Themis Z TEM (FEI, Thermo Fisher Scientific) at an accelerating voltage of 300 kV. An image corrector (CETCOR, hexapole‐type Cs‐corrector), probe corrector (DCOR, hexapole‐type Cs‐corrector), and Ceta2 camera (4k × 4k pixels, 16‐bit dynamic range, 40 frames per second) were used for in situ TEM. The in situ TEM data were analyzed using FIJI software and custom MATLAB codes.

For in situ heating TEM, CdSe nanosheets were loaded on heating chips (Protochips). The data were collected with a Tecnai G2 F20 TWIN TMP TEM (FEI, Thermo Fisher Scientific) at an operating voltage of 200 kV. An in situ heating holder (Fusion Select, Protochips) was used to control the temperature of the samples during TEM measurements.

### Material Characterization

The crystal structures of the nanocrystals were determined through XRD (Miniflex 600B, Rigaku). XPS was performed using an ESCALAB 250Xi spectrometer (Thermo Fisher Scientific) with a monochromatic Al K*α* X‐ray source (1486.6 eV). The temperature‐dependent mass change of the samples was determined by TGA (Auto Q500, TA Instrument).

### Ex Situ Electron‐Beam Irradiation Experiment

The effect of electron‐beam irradiation on the CdSe nanosheets was investigated using electron‐beam surface‐treatment equipment (CEBI‐200‐2250, Korens RTX) installed at the Cooperative Center for Research Facilities (CCRF) of Daegu Gyeongbuk Institute of Science & Technology (DGIST). Samples were prepared by depositing the CdSe nanosheets on a Ti foil, which has high electron permeability. The samples were placed parallel to the electron‐beam‐generating tube (Figure S6, Supporting Information). The dose rate of electron‐beam irradiation was regulated by controlling the operating current and time. The beam was operated at 200 kV and 10 mA for 10 min.

### Ex Situ Thermal Heating Experiment

The samples were deposited on TEM grids and heated on a hotplate for 30 min. Annealing was conducted at 50, 100, 200, and 300 °C.

### Density Functional Theory Calculations

Calculations of the structures of the 2D CdSe quantum nanosheets were performed using first‐principles DFT calculations implemented in the Vienna Ab‐initio Simulation Package (VASP). Specifically, projector augmented wave (PAW) potentials and the Perdew‐Burke Ernzerhof (PBE) generalized gradient approximation (GGA) were employed. The plane cutoff energy was set at 400 eV, and Gaussian smearing was used with the width of 0.1 eV. For all CdSe nanosheets, the convergence criterion was 10^−5^ eV for electronic energy, and the Hellmann–Feynman force was converged to 10^−2^ eV Å^−1^ for the ionic relaxation. A slab model and *z*‐direction vacuum were used to simulate the CdSe nanosheets. For wurtzite‐CdSe, the slab model consists of seven Cd–Se atomic monolayers (≈1.4 nm thickness) with the (11$\bar{2}$0) surface termination and each monolayer contains one Cd and one Se atomic layers. After the reconstruction of the nonpolar (11$\bar{2}$0) surface, supercell structures were created by forming Se defects in the wurtzite‐CdSe structures obtained by the surface reconstruction. For zinc blende‐CdSe, the slab model consists of 7 atomic monolayers, and each monolayer was a combination of one Cd and one Se atomic layers. After the (001) surface was reconstructed in the same way as described above, Se defects were formed to create supercell structures. The surface reconstruction processes were conducted by following the modified methods of previous works.^[^ ^76^, ^77^ ^]^ Supercell structures of wurtzite‐CdSe nanosheets with Se defects (Figures S9 and S10, Supporting Information) and zinc blende‐CdSe nanosheets with Se defects (Figure S11, Supporting Information) were calculated with Monkhorst‐Pack 4 × 3 × 1 k‐points and 4 × 4 × 1 k‐points, respectively. To consider the computational cost and accuracy of the energy outcome, it was identified that a vacuum space greater than 10 Å along the *z*‐axis was the optimal value for the slab model. The distance between supercell structures was far enough that there was no electronic interference between them. In the wurtzite‐CdSe nanosheets, all atoms used in the calculation were not fixed, except for the atoms in the middle layer. By contrast, all atoms in other supercells were flexible. The Se vacancy formation energy was calculated to demonstrate that Se defects can be caused by electron beam irradiation. The vacancy formation energy (*E_f_*) was calculated as per the Equation 1.

(1) $$E_{f}=E_{\mathrm{vac}}-\left(E_{\mathrm{bulk}}-\mu_{x}\right)$$

where *E*_vac_ is the energy of supercells with defects and *E*_bulk_ is the energy of original structure before the formation of defects. The chemical potential µ_x_ of the Se atom was considered as the energy of the isolated atom.^[^ ^78^ ^]^

### Calculation of Maximum Electron Transfer Energy

The maximum transferable energy from the electron beam depending on acceleration voltage was calculated by using an equation from the previous work.^[^ ^62^ ^]^ Incident electrons were assumed to be scattered elastically, and the nucleus assumed to comprise the masses of all scattering atoms.

## Conflict of Interest

The authors declare no conflict of interest.

## Supporting information

Supporting Information

Supplemental Movie 1

Supplemental Movie 2

Supplemental Movie 3

Supplemental Movie 4

Supplemental Movie 5

Supplemental Movie 6

## Data Availability

The data that support the findings of this study are available in the supplementary material of this article.
