# Supplementary material for: Direct Observation of Off‐Stoichiometry‐Induced Phase Transformation of 2D CdSe Quantum Nanosheets
Source: Adv Sci (Weinh). 2023 Jan 13;10(7):2205690. doi: 10.1002/advs.202205690 (PMC9982559; doi:10.1002/advs.202205690)
Supplement: Supplementary file 1 — Supporting Information [file ADVS-10-2205690-s003.pdf]

ADVANCED SCIENCE
Open Access

Supporting Information

for *Adv. Sci.*, DOI 10.1002/advs.202205690

Direct Observation of Off-Stoichiometry-Induced Phase Transformation of 2D CdSe Quantum Nanosheets

Hyeonjong Ma, Dongjun Kim, Soo Ik Park, Back Kyu Choi, Gisang Park, Hayeon Baek, Hyocheol Lee, Hyeongseoung Kim, Jong-Sung Yu, Won Chul Lee, Jungwon Park and Jiwoong Yang**

Supporting Information

Direct Observation of Off-Stoichiometry-Induced Phase Transformation of 2D CdSe Quantum Nanosheets

Hyeonjong Ma†, Dongjun Kim†, Soo Ik Park, Back Kyu Choi, Gisang Park, Hayeon Baek, Hyocheol Lee, Hyeongseoung Kim, Jong-Sung Yu, Won Chul Lee, Jungwon Park, and Jiwoong Yang**

H. Ma, S. I. Park, G. Park, H. Lee, H. Kim, Prof. J.-S. Yu, Prof. J. Yang
Department of Energy Science and Engineering, Daegu Gyeongbuk Institute of Science and Technology (DGIST), Daegu 42988, Republic of Korea
*Email: jiwoongyang@dgist.ac.kr

D. Kim, B. K. Choi, H. Baek, Prof. J. Park
Center for Nanoparticle Research, Institute for Basic Science (IBS), Seoul 08826, Republic of Korea
School of Chemical and Biological Engineering, and Institute of Chemical Processes, Seoul National University, Seoul 08826, Republic of Korea
*Email: jungwonpark@snu.ac.kr

Prof. W. C. Lee
Department of Mechanical Engineering, BK21 FOUR ERICA-ACE Center, Hanyang University, Ansan, Gyeonggi 15588, Republic of Korea

Prof. J.-S. Yu, Prof. J. Yang
Energy Science and Engineering Research Center, Daegu Gyeongbuk Institute of Science and Technology (DGIST), Daegu 42988, Republic of Korea

Prof. J. Park
Institute of Engineering Research, College of Engineering, Seoul National University, Seoul 08826, Republic of Korea
Advanced Institute of Convergence Technology, Seoul National University, Suwon-si, Gyeonggi-do 16229, Republic of Korea

† These authors contributed equally to this work.

Keywords: phase transformation, in-situ transmission electron microscopy, stoichiometry, quantum nanosheets, two-dimensional nanocrystals

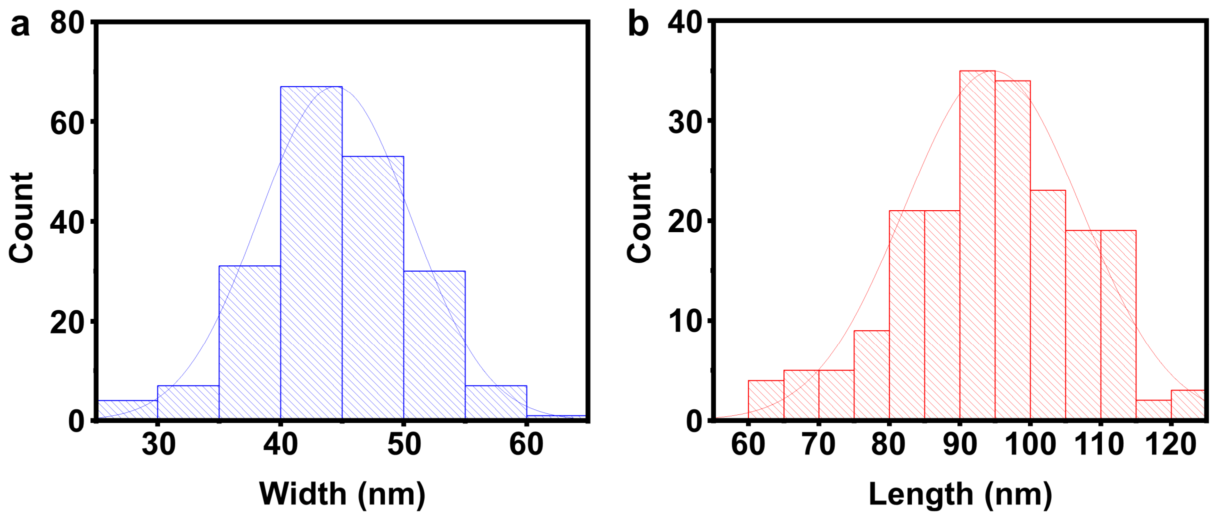

Figure S1. Histograms showing the distribution of the a) width and b) length of the wurtzite-CdSe nanosheets (n = 200).

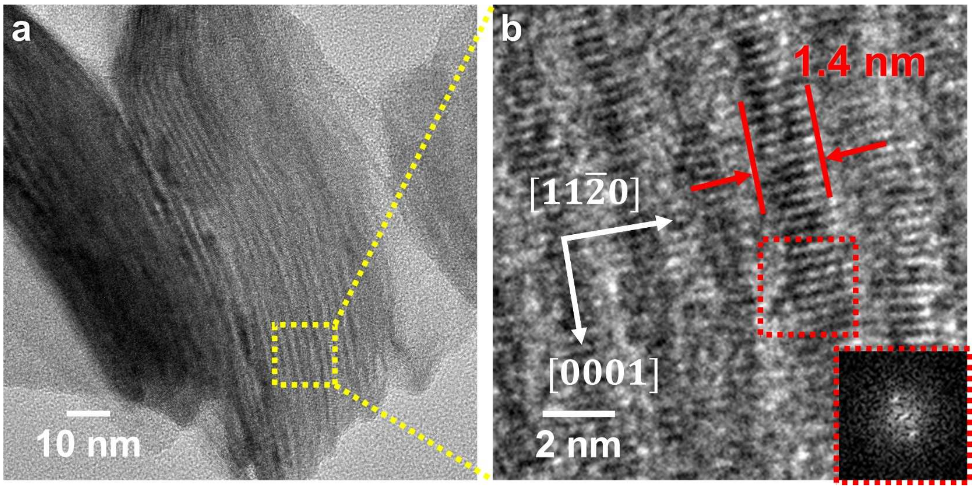

Figure S2. TEM analysis results for wurtzite-CdSe nanosheets. a) Low-magnification and b) high-resolution TEM images showing the side view of the CdSe nanosheets in the lamellar assembly. The inset shows the FFT pattern. To measure the thickness of CdSe nanosheets, we intentionally assembled them using short-chain (C8) octylamine ligands.

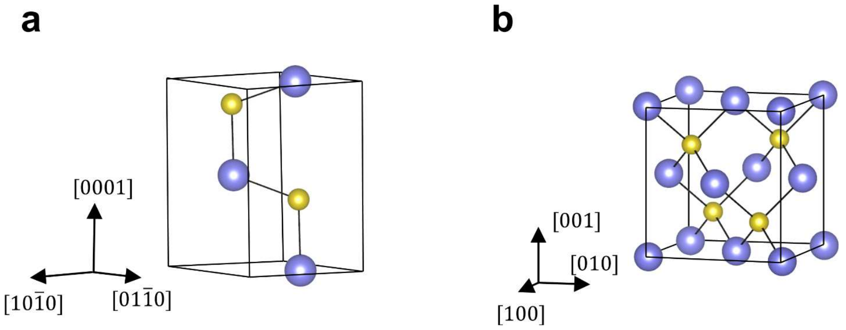

Figure S3. Schematics showing the unit cells of a) wurtzite- and b) zincblende-CdSe. The atoms with blue and yellow colors represent Cd and Se atoms, respectively.

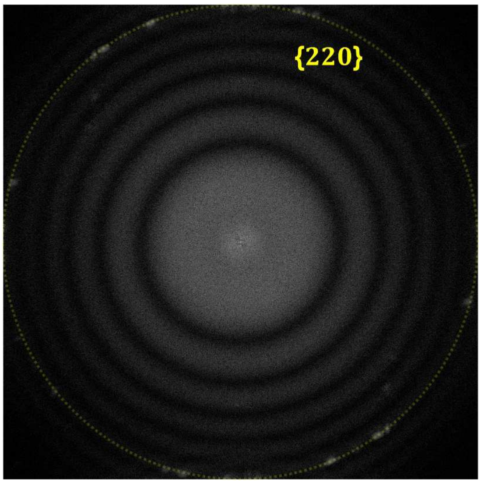

Figure S4. FFT pattern from the entire TEM image shown in Figure 1e.

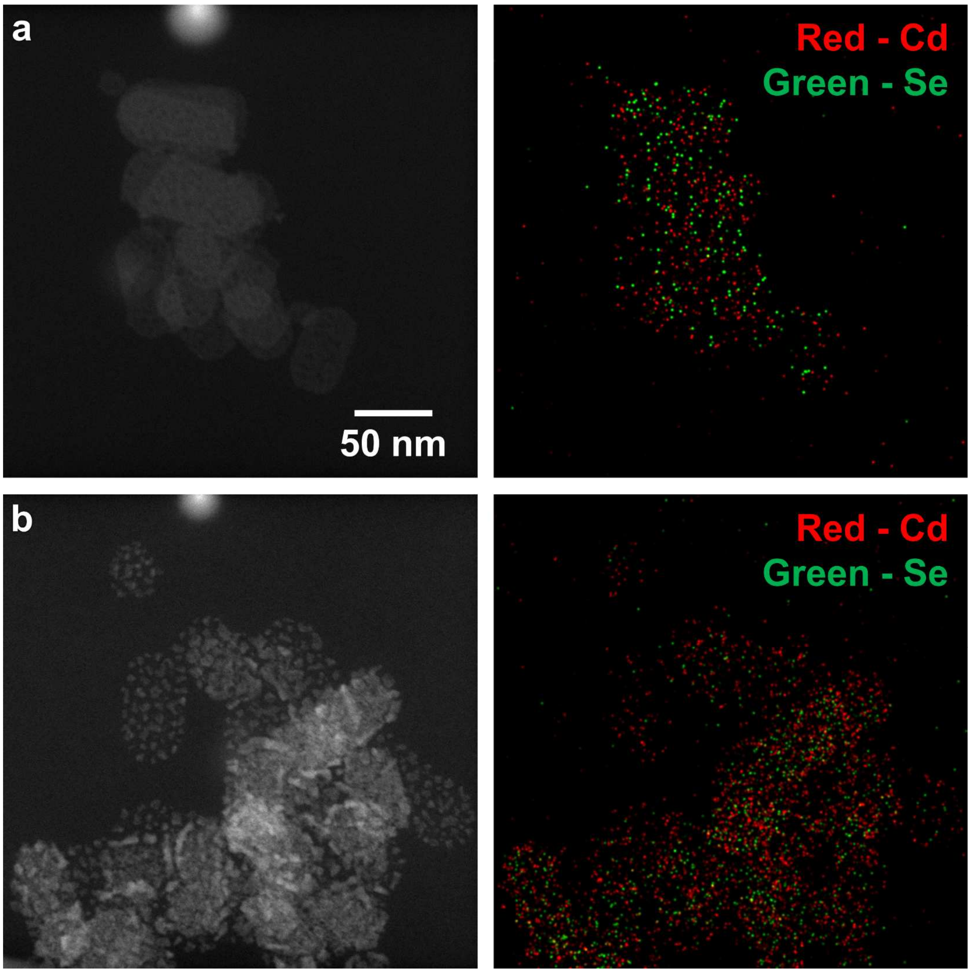

Figure S5. STEM images (left) and EDS mapping images (right) of CdSe nanosheets a) before and b) after the phase transformation.

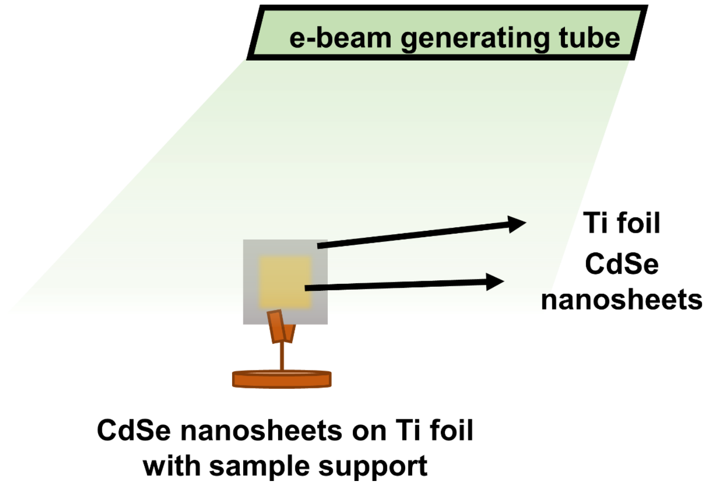

Figure S6. Schematic showing the experimental setup of the electron-beam surface-treatment system (CEBI-200-2250). The electron-beam is irradiated from the e-beam generating tube to the sample (CdSe nanosheets) on Ti foil.

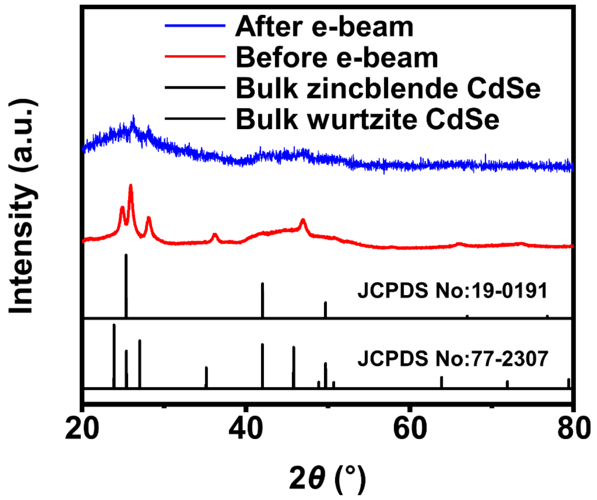

Figure S7. XRD patterns of CdSe nanosheets before and after electron-beam irradiation using electron-beam surface-treatment system (CEBI-200-2250).

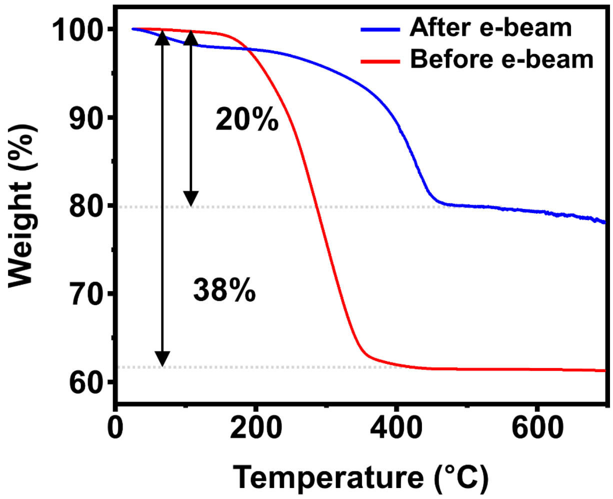

Figure S8. Thermogravimetric curves of CdSe nanosheets before and after electron-beam irradiation using electron-beam surface-treatment system (CEBI-200-2250).

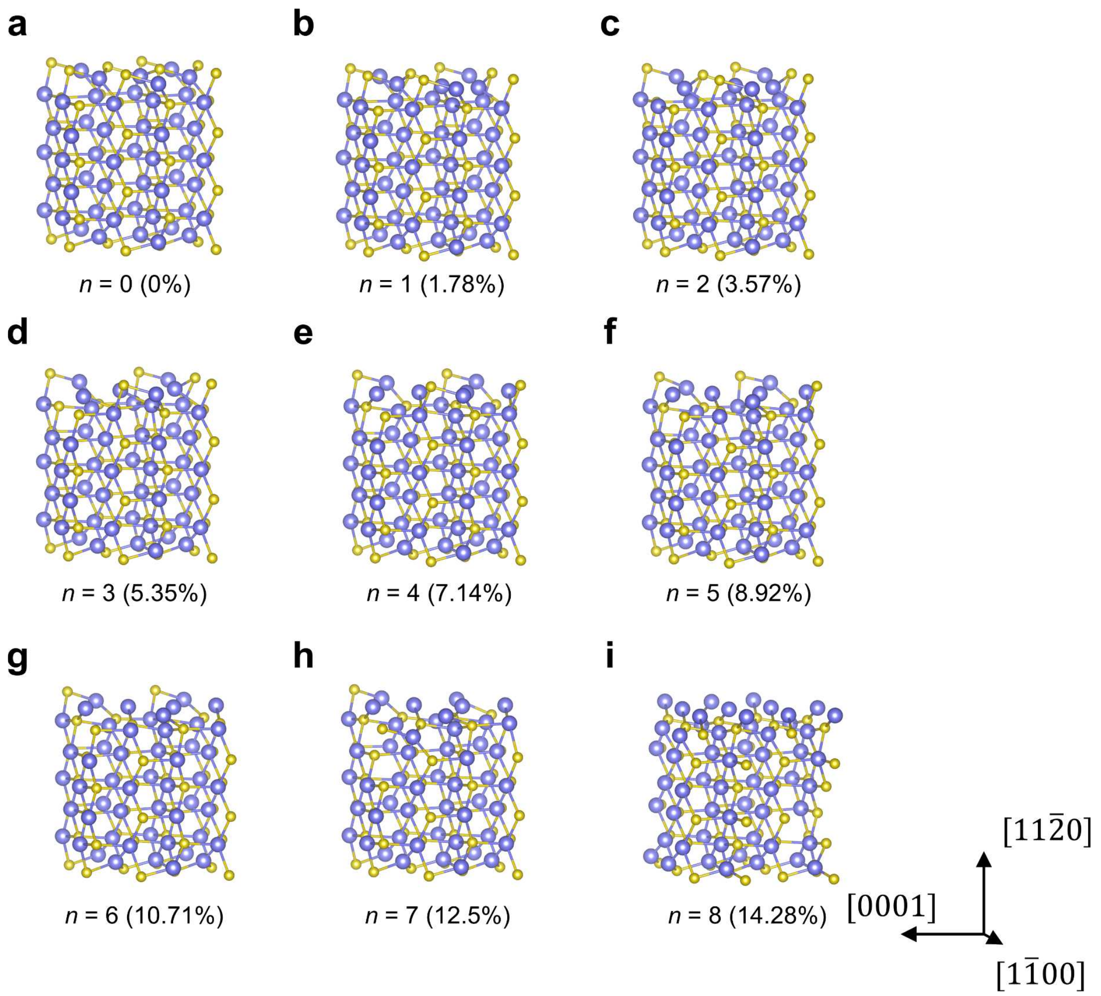

Figure S9. Supercell structures of wurtzite-CdSe nanosheets with Se defects on the surface after relaxation. *n* = the number of Se defects. The corresponding Se defect concentrations are represented together. The atoms with blue and yellow colors represent Cd and Se atoms, respectively.

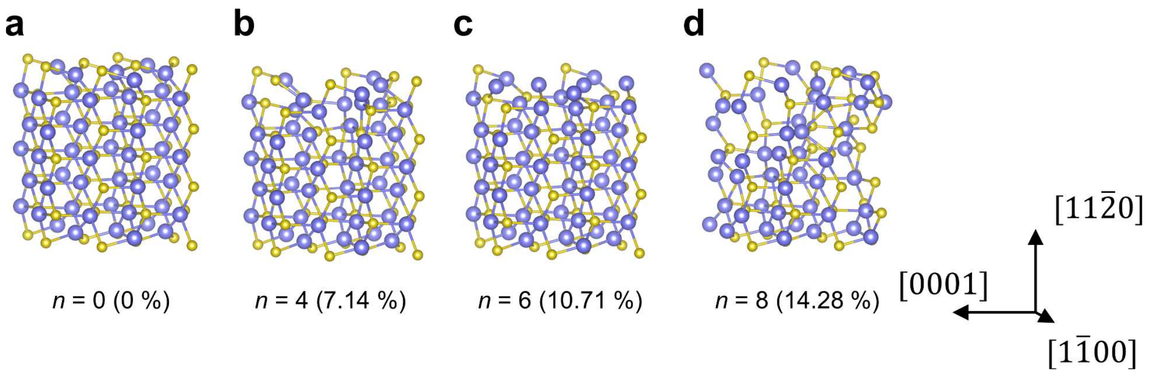

Figure S10. Supercell structures of wurtzite-CdSe nanosheets with Se defects along the vertical direction after relaxation. *n* = the number of Se defects. The corresponding Se defect concentrations are represented together. The atoms with blue and yellow colors represent Cd and Se atoms, respectively.

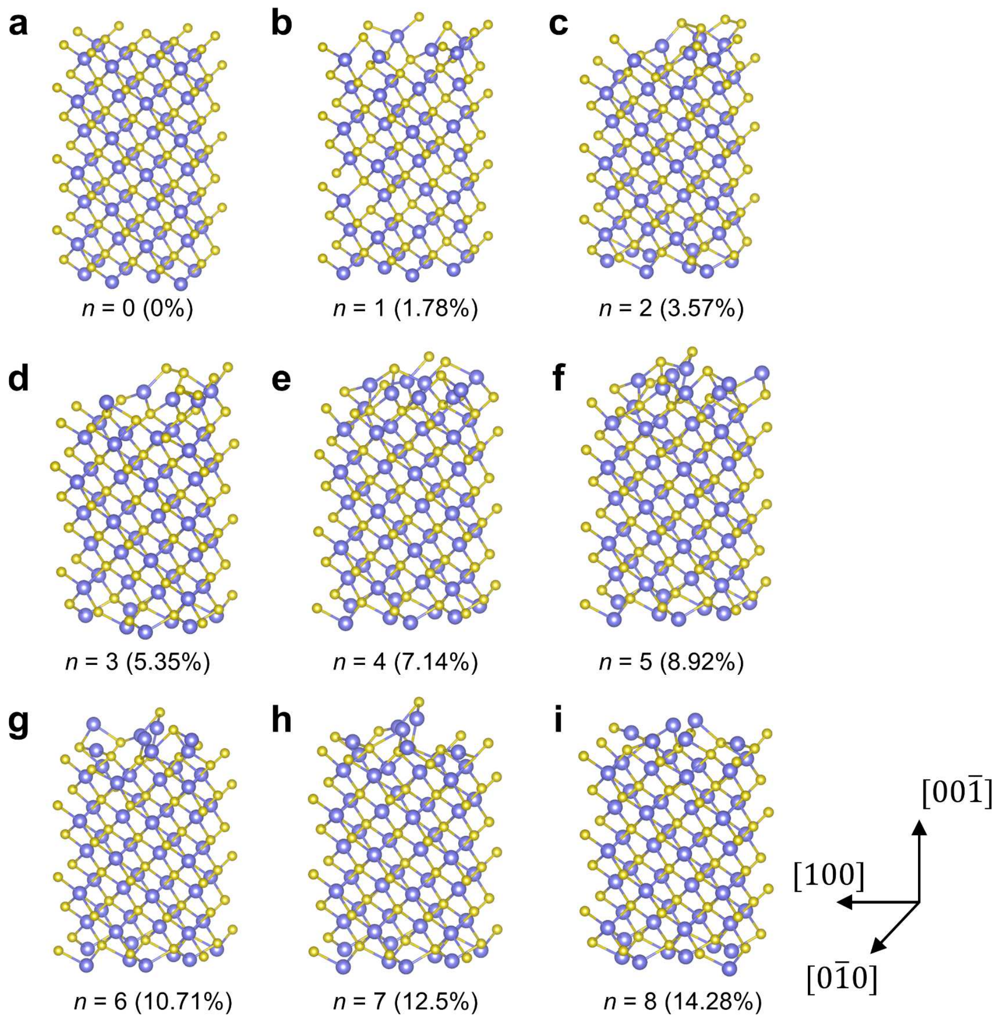

Figure S11. Supercell structures of zincblende-CdSe nanosheets with Se defects on the surface after relaxation. *n* = the number of Se defects. The corresponding Se defect concentrations are represented together. The atoms with blue and yellow colors represent Cd and Se atoms, respectively.

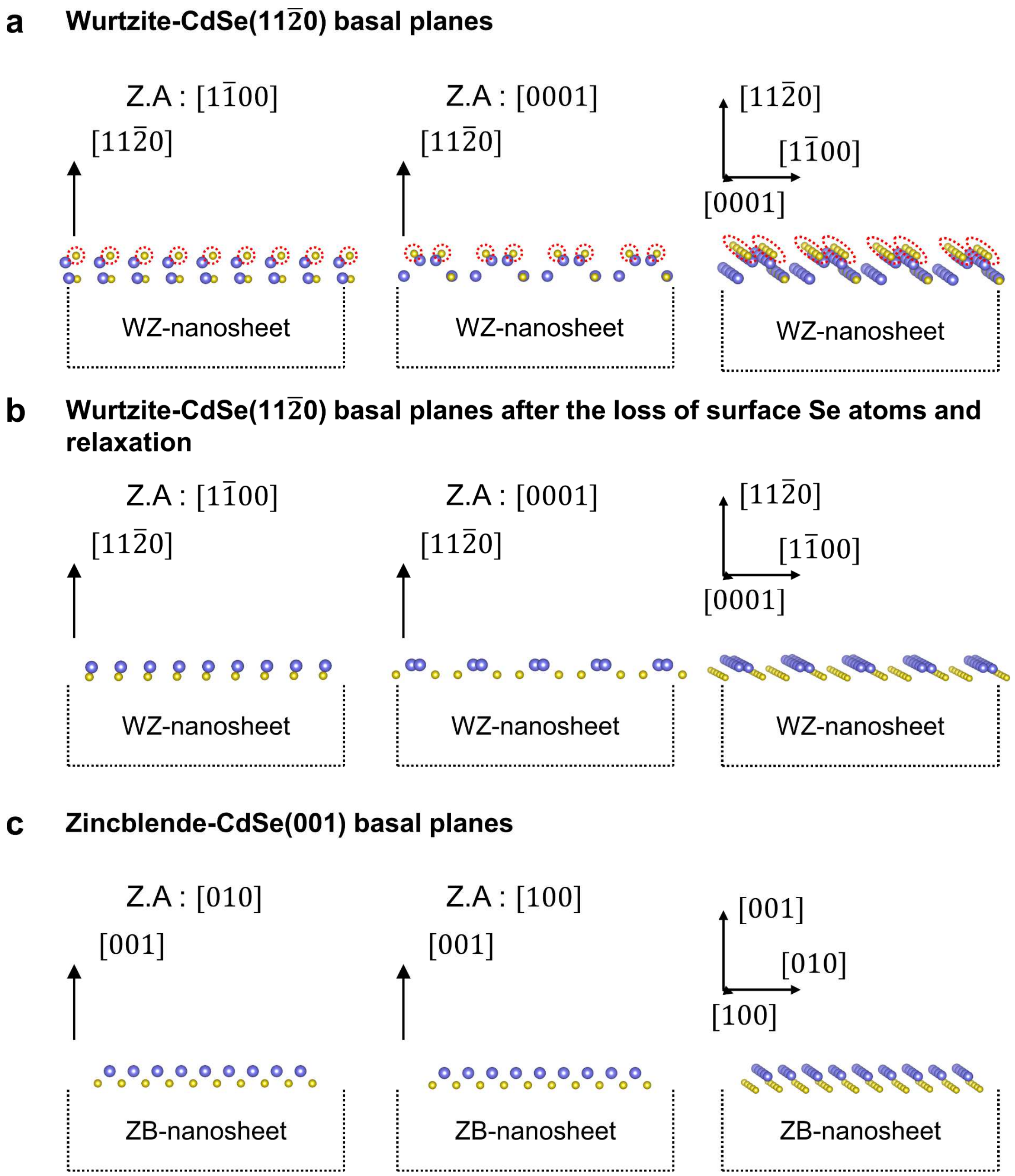

Figure S12. Surface structures of a) wurtzite-CdSe(11$\bar{2}$0) basal planes of 2D wurtzite-CdSe nanocrystals, b) wurtzite-CdSe(11$\bar{2}$0) basal planes of 2D wurtzite-CdSe nanocrystals after the loss of surface Se atoms and relaxation, and c) zincblende-CdSe(001) basal planes of 2D zincblende-CdSe nanocrystals. The atoms with blue and yellow colors represent Cd and Se atoms, respectively. The red dotted circles in Figure S12a represent Se atoms to be removed.

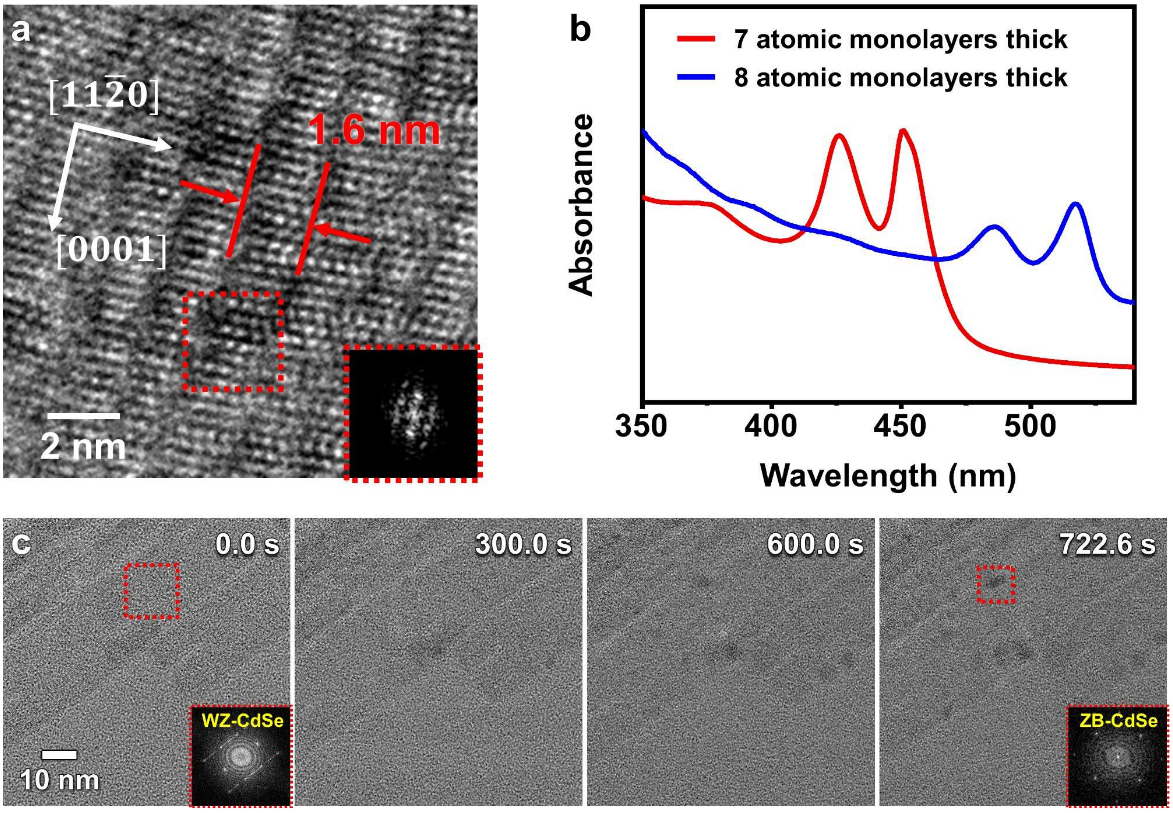

Figure S13. a) TEM image of the side view of thicker CdSe nanosheets, showing the thickness of ~1.6 nm (corresponding to 8 atomic monolayers) along [11$\bar{2}$0] direction. The inset shows the FFT pattern. To measure the thickness, the CdSe nanosheets were intentionally assembled. b) Absorption spectra of wurtzite-CdSe nanosheets with the two different thicknesses used in this study. c) Time-series TEM images showing the phase transformation of of thicker nanosheets.

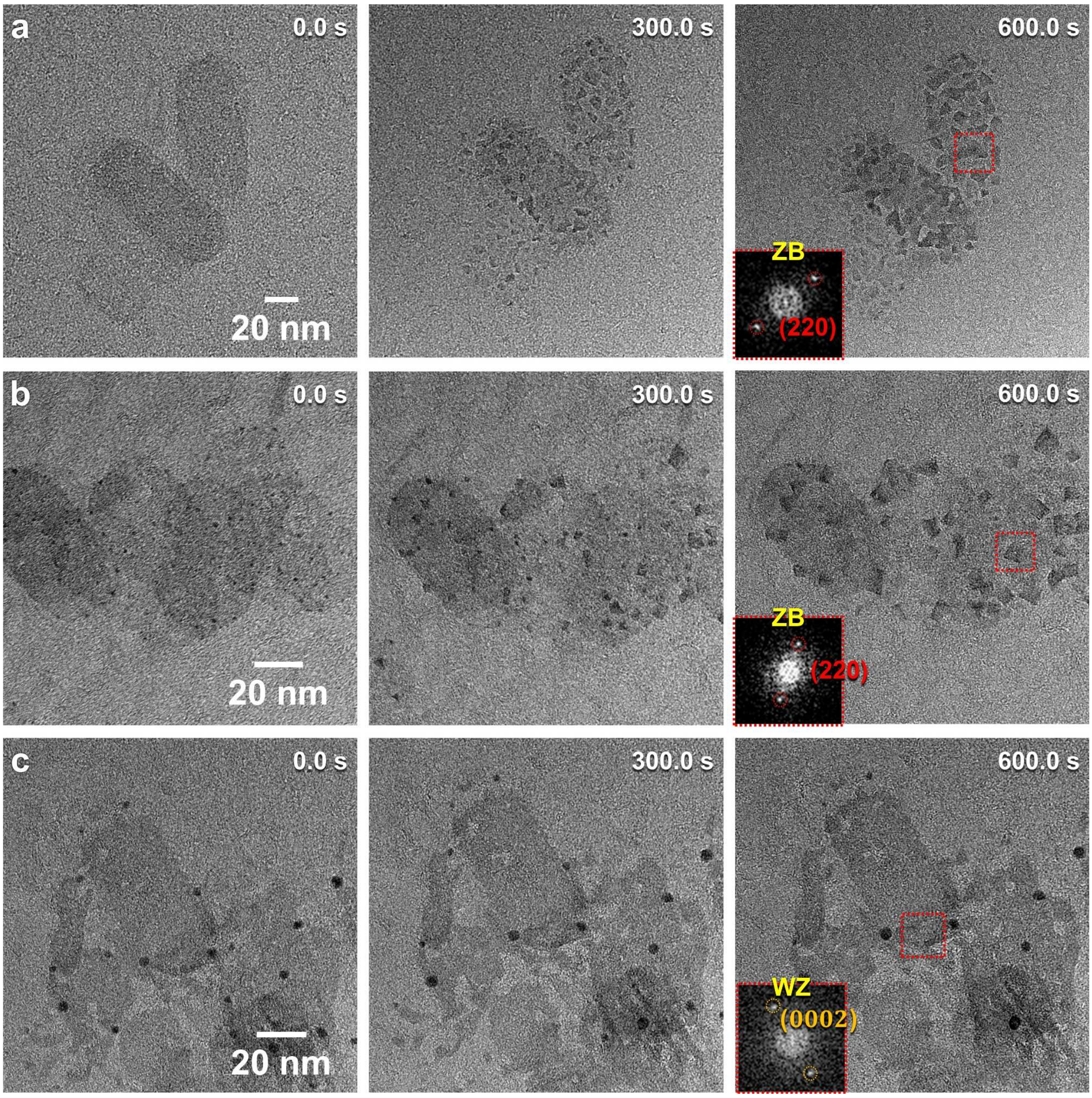

Figure S14. Time-series TEM images showing phase transformation of CdSe nanosheets at a) 100, b) 200, and c) 300 °C. Inset images represent FFT patterns of region with red dotted boxes.

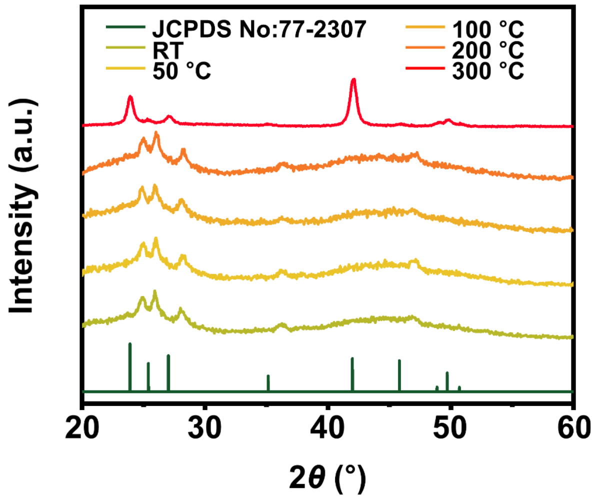

Figure S15. XRD patterns of wurtzite-CdSe nanosheets annealed at different temperatures (50–300 °C) for 30 min.

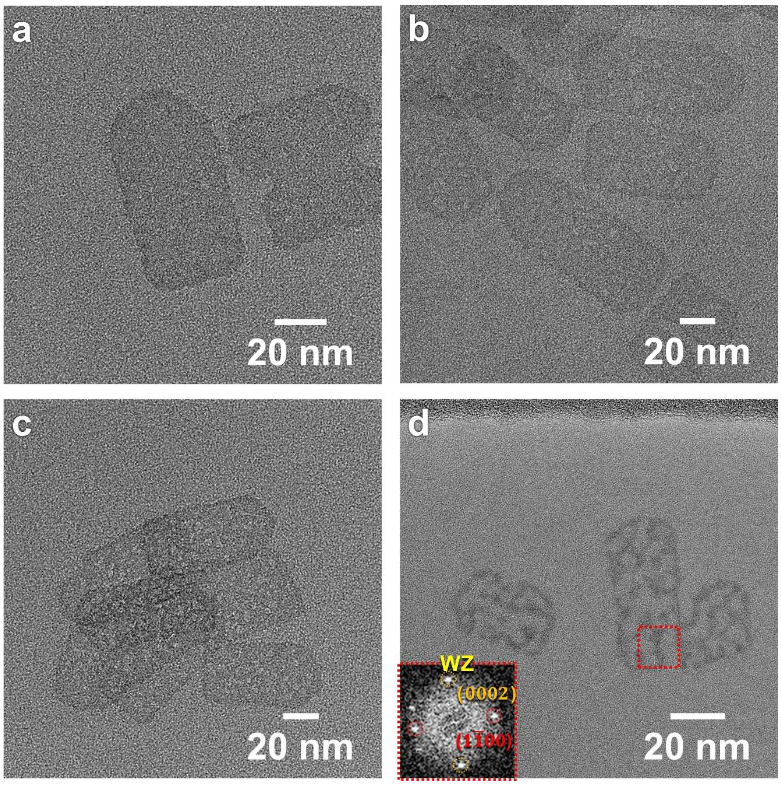

Figure S16. TEM images of CdSe nanosheets after ex-situ thermal treatment (annealing) at a) 50 °C, b) 100 °C, c) 200 °C, and d) 300 °C for 30 min.

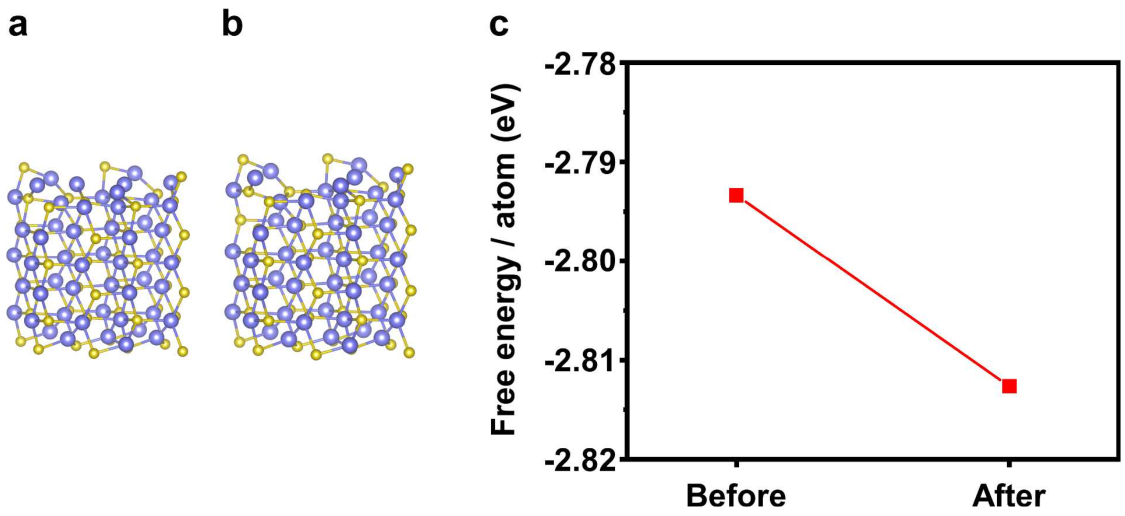

Figure S17. Supercell structures of wurtzite-CdSe nanosheets with the Se defect concentration of 8.9% (corresponding to *n* = 5 in Figure S9) a) before and b) after the Cd defect formation. c) Free energy change per atom in the wurtzite-CdSe nanosheets with the Se defect concentration of 8.9% before and after the Cd vacancy formation. The atoms with blue and yellow colors represent Cd and Se atoms, respectively.

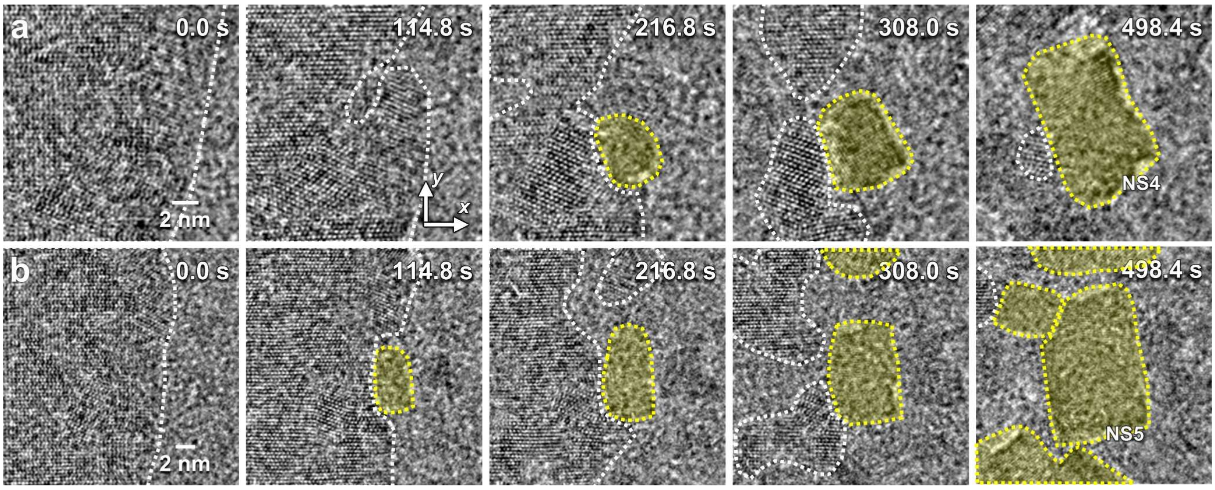

Figure S18. Time-series TEM images showing the phase transformation forming zincblende nanosheets denoted as a) NS4 and b) NS5. See Movies S3 and S4 for corresponding videos of (a) and (b), respectively.

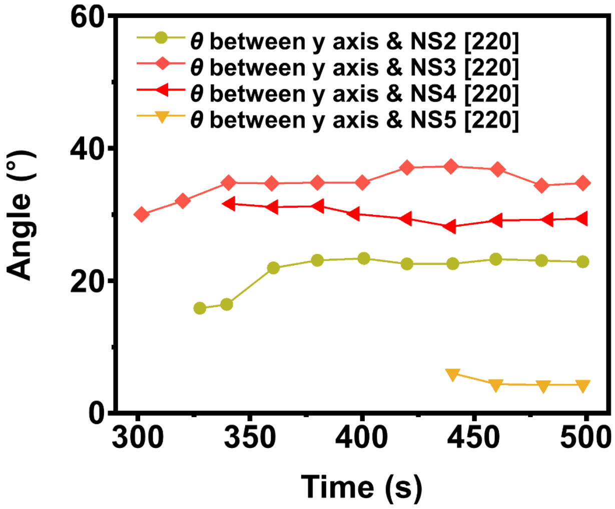

Figure S19. Change in the angle between the *y*-axis and [220] direction for four selected zincblende nanosheets (NS2–NS5) as a function of time. The *x*- and *y*-axes are represented in the second panel of Figure 3a and Figure S18.

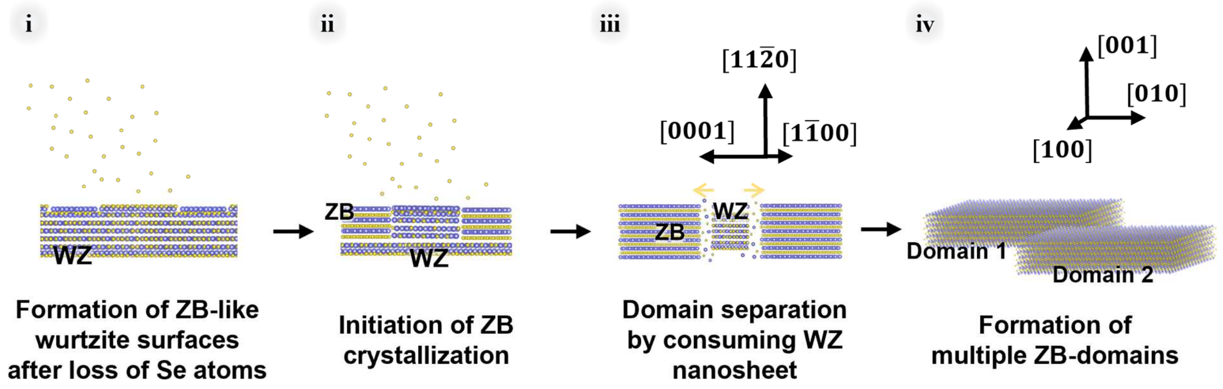

Figure S20. Schematic of the domain separation process revealed in Figure 4a. The atoms with blue and yellow colors represent Cd and Se atoms, respectively.

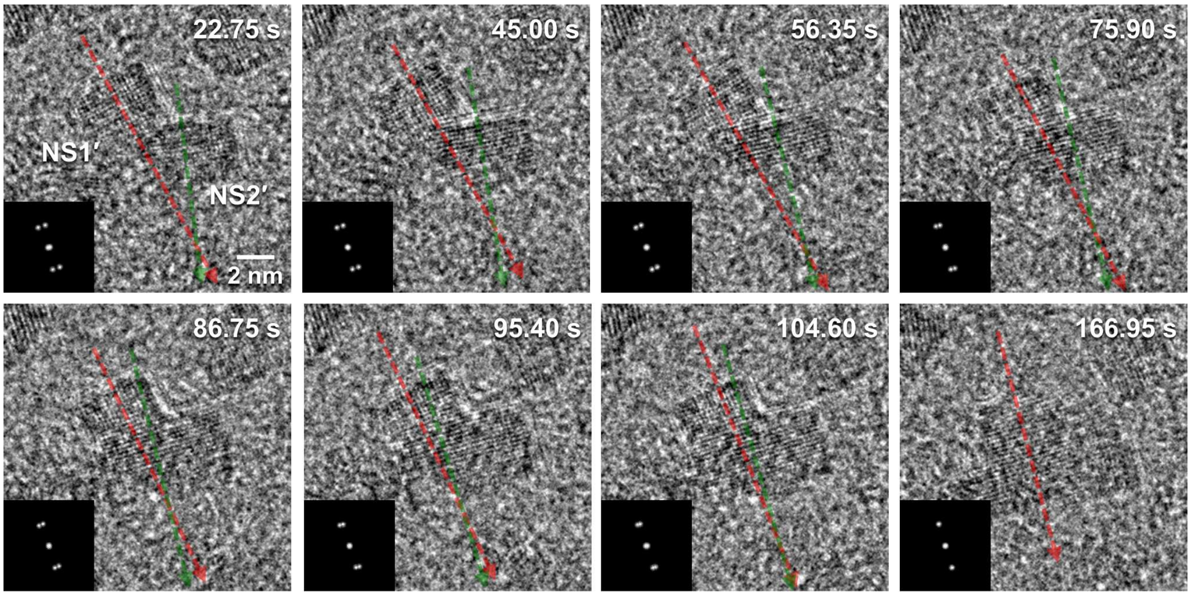

Figure S21. Time-series TEM images showing particle–particle incorporation with decreasing difference in particle orientation, as revealed in Figure 4b. The red and green dashed arrows represent the [220] directions of NS1′ and NS2′, respectively. The insets show FFT patterns of each snapshot.

Table S1. Table comparing the d-spacing (lateral direction) of wurtzite-CdSe crystals.

Plane	Bulk [nm]	Reported[S1] (2D) [nm]	Reported[S2] (1D) [nm]	This work (2D) [nm]
(0002)	0.359	0.344	0.357	0.343
$(1\bar{1}00)$	0.381	0.359		0.356

Table S2. Table comparing the d-spacing (lateral direction) of zincblende-CdSe crystals.

Plane	Bulk [nm]	Reported[S3] (2D) [nm]	Reported[S4] (0D) [nm]	This work (2D) [nm]
(220)	0.220	0.232	0.215	0.233

Information of Videos

Movie S1. In-situ TEM data showing the phase transformation of CdSe quantum nanosheets.

Movie S2. High-resolution in-situ TEM data showing the phase transformation.

Movie S3. High-resolution in-situ TEM data showing the phase transformation forming the zincblende nanosheet denoted as NS4.

Movie S4. High-resolution in-situ TEM data showing the phase transformation forming the zincblende nanosheet denoted as NS5.

Movie S5. High-resolution in-situ TEM data showing the domain separation process.

Movie S6. High-resolution in-situ TEM data showing the coalescence of zincblende nanosheets.

References

[S1] J. S. Son, X.-D. Wen, J. Joo, J. Chae, S.-I. Baek, K. Park, J. H. Kim, K. An, J. H. Yu, S. G. Kwon, S.-H. Choi, Z. Wang, Y.-W. Kim, Y. Kuk, R. Hoffmann, T. Hyeon, *Angew. Chem. Int. Ed.* **2009**, *48*, 6861.

[S2] G. Ramalingam, N. Melikechi, P. Dennis Christy, S. Selvakumar, P. Sagayaraj, *J. Cryst. Growth* **2009**, *311*, 3138.

[S3] Z. Li, X. Peng, *J. Am. Chem. Soc.* **2011**, *133*, 6578.

[S4] L. Liu, Z. Zhuang, T. Xie, Y.-G. Wang, J. Li, Q. Peng, Y. Li, *J. Am. Chem. Soc.* **2009**, *131*, 16423.
